# Factors associated with improvement in disease activity following initiation of etanercept in children and young people with Juvenile Idiopathic Arthritis: results from the British Society for Paediatric and Adolescent Rheumatology Etanercept Cohort Study

**DOI:** 10.1093/rheumatology/kev434

**Published:** 2015-12-30

**Authors:** Lianne Kearsley-Fleet, Rebecca Davies, Mark Lunt, Taunton R. Southwood, Kimme L. Hyrich

**Affiliations:** ^1^Arthritis Research UK Centre for Epidemiology, Manchester Academic Health Science Centre, The University of Manchester, Manchester,; ^2^Department of Paediatric Rheumatology, Institute of Child Health, Birmingham Children’s Hospital – NHS Trust and University of Birmingham, Birmingham and; ^3^NIHR Manchester Musculoskeletal Biomedical Research Unit, Central Manchester University Hospitals NHS Foundation Trust and University of Manchester Partnership, Manchester, UK

**Keywords:** Juvenile Idiopathic Arthritis, epidemiology, biological therapies, outcome measures, statistics

## Abstract

**Objectives.** The objectives of this study were to investigate change in disease activity, and explore factors associated with response, in children with JIA over the initial year of etanercept treatment.

**Methods.** This analysis included children with JIA starting etanercept in the British Society for Paediatric and Adolescent Rheumatology Etanercept Cohort Study. Response was assessed using change in juvenile arthritis disease activity score-71 (JADAS-71), an excellent response (ACR Pedi 90), and achieving minimal disease activity (MDA) at 1 year. Change in JADAS-71 was evaluated over time. Multivariable backward stepwise logistic regression was performed to identify factors associated with ACR Pedi 90 and MDA.

**Results.** A total of 496 children were included. Over the first year, 17 stopped due to inefficacy, 9 due to adverse events and 7 for other reasons. One child stopped for remission. At 1 year, 74, 69 and 38% reached ACR Pedi 30, 50 and 90, respectively, and 48% had achieved MDA. Independent predictors of achieving ACR Pedi 90 at 1 year included shorter disease duration [odds ratio (OR) 0.91; 95% CI: 0.85, 0.97)], no concurrent oral corticosteroid use (OR 0.48; 95% CI: 0.29, 0.80) and history of uveitis (OR 2.26; 95% CI: 1.08, 4.71). Independent predictors of achieving MDA at 1 year included younger patients (OR 0.60; 95% CI: 0.38, 0.95), and disease not treated with concurrent oral corticosteroids (OR 0.57; 95% CI: 0.35, 0.93).

**Conclusion.** Among this real-world cohort of children with severe JIA, a significant proportion of children achieved an excellent ACR Pedi response and MDA within 1 year of starting etanercept, although few clinical factors could predict this outcome.

Rheumatology key messages

- Half of children with JIA treated with etanercept achieved minimal disease activity by 1 year.
- Children with JIA who did not require corticosteroid treatment were more likely to achieve excellent response while receiving etanercept.
- Younger children with JIA were more likely to achieve excellent response on etanercept than older children.

## Introduction

JIA affects approximately 1 in 1000 children in the UK [1], with many continuing to have considerable disability related to prolonged active disease into adulthood [2]. First line therapy for children with polyarticular JIA usually includes the synthetic DMARD (sDMARD) MTX. For children who do not respond or are intolerant of MTX, biologic DMARD therapies can be prescribed. TNF was the first validated cytokine to be used as a biologic target for inflammatory arthritis [3], and etanercept was the first of these biologic anti-TNF therapies to be licensed in Europe for children with JIA [4].

Many studies looking at response to anti-TNF therapy in adults with RA have demonstrated that lower disease activity measures, lesser disability and concurrent MTX use at treatment start are associated with good treatment response, or remission [5–10]. However, similar studies in children with JIA are limited. Within small groups of children, disease outcomes and quality of life have been shown to improve on treatment with etanercept [11–13]. A common finding in the literature is that children with JIA with lower disease severity at the start of etanercept treatment are more likely to respond [14, 15].

To date, four observational studies have looked in-depth at factors associated with response in children with JIA treated with etanercept [14–17] with sample sizes ranging from 61 to 863 (supplementary Table S1, available at *Rheumatology* Online). These studies have varied to some degree in methodology including definition of the outcome. Three studies explored factors associated with a good response [14, 15, 17]. One of these also explored factors associated with non-response [17], as did a study by Quartier *et al.* [16]. Factors found to be associated in some, but not all studies, with response included age (better response among younger children), childhood health assessment questionnaire (CHAQ) (better response in those with lower CHAQ at start of etanercept) and JIA ILAR category [18] (lesser response in children with systemic JIA). Most recently, the German BiKeR register studied a large group of children with JIA (n = 863) starting etanercept therapy. They reported a number of factors associated with achieving ACR Pedi 70 response at 6 months; lower CHAQ, higher ESR, no steroid use at start of therapy, non-systemic JIA and younger age [14]. A fifth study looking at treatment survival also found systemic JIA, chronic anterior uveitis (CAU), and inefficacy of MTX to be associated with discontinuation of etanercept therapy [19]. Despite these published studies, there remains no clear consensus on whether clinical factors are associated with response. Replication of work in different cohorts of patients, and different countries, where access to and use of biologic therapies may differ, is important in order to describe and understand the spectrum of response being observed with etanercept. Consistencies in findings, particularly with respect to factors associated with response, may warrant further investigation to understand causal pathways. Therefore the aims of this study were to investigate change in disease activity in children in the UK with severe JIA over the initial year of treatment with etanercept and explore factors associated with response over this same period.

## Methods

### Study design

The British Society for Paediatric and Adolescent Rheumatology Etanercept Cohort Study (BSPAR-ETN) is an ongoing national prospective observational study established in 2004. It was approved by the West Midlands Research Ethics Committee with the aim of collecting long-term outcome data on children with JIA starting etanercept treatment. Forty-two UK centres have currently been enrolled in the study. Written informed consent of the parents and patients are provided in accordance with the Declaration of Helsinki, and this includes consent for their data to be used in analyses. This analysis did not require further ethical approval to analyse the data from the BSPAR-ETN.

### Data collection

At the start of etanercept treatment, patient information was collected by a consultant or clinical research nurse via a questionnaire. This included patient demographics (age, gender), disease duration, ILAR category, past and current anti-rheumatic therapies including any prior biologics, history of CAU and current disease activity; JIA Core Disease Outcome Variables [20] [active joint count (AJC), limited joint count, ESR, CRP, physician global assessment of disease (PGA), parent/patient global assessment of wellbeing (PtGE), CHAQ] and pain visual analogue scale (VAS). The same data were then collected at follow-up intervals at 6 and 12 months and then annually thereafter.

### Statistical analysis

This analysis was restricted to children treated with etanercept as their first biologic with follow-up records available at baseline and 1 year by 10 September 2014. Only patients who satisfied the ILAR criteria for JIA were included; patients with a missing ILAR were excluded from this analysis. Patients in minimal disease activity (MDA) [21] at start of etanercept therapy were excluded from the analysis as it was not clear whether this related to baseline (e.g. pre-etanercept) or current (post-etanercept) disease activity.

Patients who stopped etanercept therapy within the first year were classified as non-responders, unless they stopped for remission, in which case they were then classified as responders. Response was assessed in three ways: change in juvenile arthritis disease activity score-71 (JADAS-71); ACR Pedi 90; and, MDA at 1 year. Change in JADAS-71 was evaluated over time using a longitudinal linear regression model with random effects. This allowed for within-patient correlation of JADAS-71 over the multiple measurements. JADAS-71 is a composite disease activity score for patients with JIA that allows disease activity to be measured at a single time point, comprising AJC, PGA, PtGE and ESR [22]. The ACR Pedi criteria were originally defined in 1997 [20]. The ACR Pedi 90 is defined as at least a 90% improvement in three of the six JIA core disease outcome variables, with no more than one of the variables worsening by > 30%. As a variable, ACR Pedi 90 allows change in disease activity over time to be assessed; however it does not highlight disease status at a single time point. MDA is a useful concept in clinical and epidemiological research, assessing disease status at a single time point, as it represents a more realistic goal for patients with JIA rather than remission. For patients with persistent oligoarthritis, MDA was defined as PGA ⩽ 2.5 cm and 0 AJC. For all other patients, MDA was defined as PGA ⩽ 3.4 cm, PtGE ⩽ 2.1 cm and AJC ⩽ 1. Patients with enthesitis-related arthritis were excluded from the MDA analysis as the score had not been validated in these patients [21].

Univariable and multivariable logistic regression were performed to identify factors associated with an excellent response (ACR Pedi 90 [20]) and achieving MDA [21] at 1 year. Patients who stopped etanercept due to adverse events or for other reasons were excluded from the prediction model to minimize competing risks, as initial response prior to stopping therapy was not captured. Model building followed a backward stepwise process (two-sided 5% significance level). Variables available for selection were chosen *a priori* for biological value. Multiple imputation was used to account for missing disease activity data, using the ice package in Stata [23], with 20 iterations. Variables included gender, age at start of etanercept, disease duration, discontinuation of etanercept, ILAR category, concomitant oral corticosteroids, concomitant MTX, baseline history of CAU, disease activity measures at start of etanercept and at 1 year (AJC, limited joint count, PGA, PtGE, CHAQ, pain VAS, ESR, CRP, JADAS-71), and MDA at 1 year. All continuous variables were transformed to a normal distribution for the imputation. All analysis was performed in Stata (StataCorp. 2013. Stata Statistical Software: Release 13. StataCorp LP, College Station, TX, USA).

## Results

### Baseline characteristics

A total of 496 children were included in this analysis with baseline and 1 year follow-up available. The majority of patients were female (67%) and the most common ILAR subtype was polyarthritis RF negative (36%). The median age at the start of treatment was 11.2 [inter-quartile range (IQR): 7.6–13.7] years, with a median disease duration of 3 (IQR: 2–7) years. Over half of the patients were on concomitant MTX (55%), and 28% were using concurrent oral corticosteroids at start of treatment, 56% in children with systemic JIA and 24% in those with non-systemic JIA. Median CHAQ was 1.3 (IQR: 0.6–2.0), and median JADAS-71 was 16.9 (IQR: 12.0–24.5) (Table 1).

**Table 1:** Characteristics of children with JIA starting treatment with etanercept (N = 496), including measures at 1 year

Characteristics	Baseline	1 year
Female, n (%)	332 (67), [N = 496]	
Age, median (IQR), years	11.2 (7.6–13.7) [N = 496]	
Disease duration, median (IQR), years	3 (2–7) [N = 488]	
Concurrent MTX use, n (%)	275 (55) [N = 496]	207 (42) [N = 496]
Oral steroid use, n (%)	141 (28) [N = 496]	105 (23) [N = 466]
Systemic arthritis only, n (%)	41 (56)	25 (37)
Excluding systemic arthritis, n (%)	100 (24)	80 (20)
History of chronic anterior uveitis, n (%)	48 (10) [N = 496]	
ILAR subgroup, n (%)	[N = 496]	
Systemic arthritis^a^	73 (15)	
Persistent oligoarthritis	12 (2)	
Extended oligoarthritis	81 (16)	
Polyarthritis RF negative	181 (36)	
Polyarthritis RF positive	43 (9)	
PsA	36 (7)	
Enthesitis-related arthritis	35 (7)	
Undifferentiated arthritis	35 (7)	
Disease activity, median (IQR)		
Active joint count (0–72)	6.0 (3.0–12.0) [N = 445]	0.0 (0.0–2.0) [N = 451]
Limited joint count (0–72)	5.0 (2.5–11.0) [N = 428]	1.0 (0.0–5.0) [N = 435]
Physician global of disease (0–10 cm)	4.2 (2.7–6.0) [N = 305]	0.9 (0.0–2.0) [N = 344]
Parent/patient global of well-being (0–10 cm)	5.0 (2.6–7.0) [N = 330]	1.3 (0.2–3.9) [N = 349]
Childhood health assessment questionnaire (0–3)	1.3 (0.6–2.0) [N = 243]	0.4 (0.0–1.1) [N = 341]
Pain VAS (0–10 cm)	4.8 (2.7–7.0) [N = 296]	1.6 (0.2–4.0) [N = 333]
ESR, mm/h	20.0 (7.0–47.0) [N = 395]	7.0 (4.0–17.0) [N = 345]
CRP, mg/l	11.0 (5.0–42.0) [N = 390]	5.0 (3.0–7.0) [N = 348]
Juvenile arthritis disease activity score-71	16.9 (12.0–24.5) [N = 218]	3.8 (0.8–9.0) [N = 237]

^a^22 (38%) of the 58 systemic patients with available data reported active extra-articular systemic features at start of etanercept therapy.

n: number; N: number of available records; IQR: interquartile range.

### Over 1 year of treatment with etanercept

Of the 496 children included in this analysis, 17 (3%) stopped etanercept due to inefficacy, 9 (2%) due to adverse events and 7 (1%) for other reasons. One child stopped for remission within the first year. At 1 year, 74, 69, 56 and 38% had reached ACR Pedi 30, 50, 70 and 90, respectively, and 48% had achieved MDA. Median JADAS-71 at baseline was 16.6 (IQR: 12.0–24.1). At 1 year this decreased to 3.7 (IQR: 0.6–9.3; P < 0.001). When patients were grouped by ILAR category, JADAS-71 improved after 1 year of treatment in all ILAR subtypes, although this did not reach statistical significance in patients with persistent oligoarticular JIA (Table 2).

**Table 2:** Disease activity measures after 1 year of etanercept treatment in children with juvenile idiopathic arthritis, by ILAR subgroup (n = 496), using imputed data

JIA subtype	JADAS-71, median (IQR) Baseline	JADAS-71, median (IQR) 1 year	ACR Pedi 30, %	ACR Pedi 50, %	ACR Pedi 70, %	ACR Pedi 90, %	MDA^a^, % [n = 461]
All patients (n = 496)	16.6 (12.0–24.1)	3.7 (0.6–9.3)**	74	69	56	38	48
Systemic (n = 73)	22.3 (15.5–33.8)	5.3 (1.1–11.9)**	69	64	48	27	37
Persistent oligoarticular (n = 12)	7.2 (3.8–10.2)	3.1 (0.7–5.6)	80	78	70	62	74
Extended oligoarticular (n = 81)	14.6 (9.0–19.2)	3.7 (1.0–8.0)**	76	70	58	38	46
Polyarticular RF negative (n = 181)	17.0 (11.7–24.6)	3.2 (0.7–9.3)**	76	71	60	39	51
Polyarticular RF positive (n = 43)	17.3 (13.8–22.9)	6.3 (0.7–13.5)**	62	56	43	37	39
Psoriatic (n = 36)	17.5 (12.4–24.8)	6.1 (1.0–14.3)*	60	58	50	39	48
Enthesitis-related (n = 35)	17.2 (12.4–22.0)	3.0 (0.5–5.9)**	89	84	68	45	-
Undifferentiated (n = 35)	17.1 (11.6–28.5)	1.7 (0.04–6.7)**	83	79	60	45	56

^a^Patients with enthesitis-related arthritis were excluded from the MDA response.

*P < 0.05.

**P < 0.001.

n: number; JADAS: juvenile arthritis disease activity score; MDA: minimal disease activity.

### Factors associated with response after 1 year of treatment with etanercept

Of the 480 patients included in the univariable analysis (which excluded those who stopped etanercept due to adverse events or for other reasons), factors associated with achieving ACR Pedi 90 at 1 year included younger age, shorter disease duration, disease that did not require concurrent oral corticosteroid treatment and lower CHAQ. Only younger age and no concurrent oral corticosteroid use were associated with achieving MDA at 1 year (Table 3). However, in the multivariable analysis, independent predictors of achieving ACR Pedi 90 at 1 year included shorter disease duration [odds ratio (OR) 0.91; 95% CI: 0.85, 0.97], disease that did not require concurrent oral corticosteroid treatment (OR oral corticosteroid use 0.48; 95% CI: 0.29, 0.80), and history of CAU (OR 2.26; 95% CI: 1.08, 4.71). In addition, younger patients had increased odds of achieving MDA at 1 year compared with older patients (OR 0.60 age ⩾9 years old compared with <9 years; 95% CI: 0.38, 0.95), as well as those patients with disease that did not require concurrent oral corticosteroid treatment (OR oral corticosteroid use 0.57; 95% CI: 0.35, 0.93). The largest fraction of missing information lay between 0.14 and 0.59 for all odds ratios.

**Table 3:** Univariable and multivariable analysis of factors associated with an excellent response and minimal disease activity 1 year after commencing etanercept

	ACR Pedi 90 at 1 year(N = 480)		Minimal disease activity at 1 year^a^ (N = 447)	
Baseline characteristics at start of etanercept therapy	Univariable analysis, odds ratio (95% CI)	Multivariable analysis, odds ratio (95% CI)	Univariable analysis, odds ratio (95% CI)	Multivariable analysis, odds ratio (95% CI)
Female	0.97 (0.63, 1.49)		1.05 (0.67, 1.63)	
Age, years	0.96 (0.91, 1.01)		0.95 (0.90, 1.00)	
Aged ≥9 years old	0.62 (0.41, 0.96)*		0.64 (0.41, 0.99)*	0.60 (0.38, 0.95)*
Disease duration, years	0.93 (0.87, 0.98)*	0.91 (0.85, 0.97)*	0.97 (0.92, 1.04)	
Systemic arthritis	0.56 (0.29, 1.07)		0.63 (0.35, 1.11)	
Concurrent oral corticosteroid use	0.52 (0.31, 0.85)*	0.48 (0.29, 0.80)*	0.61 (0.38, 0.98)*	0.57 (0.35, 0.93)*
Concurrent MTX use	1.27 (0.85, 1.92)		1.14 (0.75, 1.73)	
History of uveitis	2.02 (0.99, 4.10)	2.26 (1.08, 4.71)*	2.08 (0.88, 4.88)	
Disease activity				
Active joint count (0–72)	0.98 (0.96, 1.01)		0.98 (0.96, 1.00)	
Limited joint count (0–72)	0.98 (0.96, 1.00)		0.99 (0.96, 1.01)	
Physician global assessment of disease (0–10 cm)	1.00 (0.91, 1.10)		0.91 (0.82, 1.00)	
Parent/patient global assessment of well-being (0–10 cm)	0.97 (0.89, 1.07)		0.90 (0.81, 1.00)	
Childhood health assessment questionnaire (0–3)	0.70 (0.51, 0.95)*		0.72 (0.51, 1.02)	
Pain VAS (0–10 cm)	0.95 (0.87, 1.03)		0.90 (0.82, 1.00)	
ESR, mm/h	1.00 (1.00, 1.01)		1.00 (0.99, 1.00)	
CRP, mg/l	1.00 (1.00, 1.01)		1.00 (0.99, 1.00)	
Juvenile arthritis disease activity score-71	0.99 (0.97, 1.01)		0.98 (0.96, 1.00)	

^a^Patients with enthesitis-related arthritis were excluded from the minimal disease activity response.

*P < 0.05.

## Discussion

This UK study is one of the largest to look at response in children with severe JIA treated with etanercept. This analysis has demonstrated improvement in disease activity of children treated with etanercept with 38% of patients achieving an excellent response and 48% of patients achieving MDA after 1 year. Independent predictors of achieving ACR Pedi 90 at 1 year included shorter disease duration, disease that did not require concurrent oral corticosteroid treatment, and history of CAU. In addition, independent predictors of achieving MDA at 1 year included younger age and disease that did not require concurrent oral corticosteroid treatment.

Younger age has been described previously with respect to response in children with JIA treated with etanercept by the Dutch ABC register [15], in a study from Italy [17] and in the German BiKeR register [14] (Table 1). The German BiKeR study, on a large number of children (n = 863), was the only study to highlight concomitant corticosteroid use as a negative predictor of good response on etanercept [14]. Disease duration and history of CAU are novel findings in the current study for patients achieving ACR Pedi 90 at 1 year. The association with disease duration may reflect the presence of more chronic joint limitations or pain that may be less responsive to anti-TNF therapies. This association between disease duration prior to treatment and greater response on biologic therapy may be an important observation as it is something that clinicians can aim to shorten, and recent treatment strategy trials have shown the benefit of early treatment in JIA [11, 24].

The finding that presence of CAU predicted good clinical response is interesting but may also be counterintuitive given the evidence that suggests etanercept is no better than placebo at preventing relapse of CAU [25, 26]. However, this study defined response as improvements in articular symptoms only and it is possible that the articular disease was more responsive or perhaps less severe in children with a history of CAU. In addition, a previous study looking at treatment survival found CAU to be associated with discontinuation of etanercept therapy [19]. However, this outcome often cannot differentiate between stopping treatment for non-response compared with reasons of safety. There was no difference in the current study between baseline JADAS-71 of patients with and without CAU. However, there were differences in proportion of CAU patients between ILAR categories; as expected, fewer systemic JIA patients had CAU (4%), and a higher proportion of oligoarticular persistent (25%) and extended (20%) had CAU. Although this may not be an indication to choose etanercept among children with this history, it may indicate that the arthritis itself may respond well to etanercept therapy in this subgroup. Increasingly there is a trend to use alternative anti-TNF therapies in this population for the concerns regarding eye disease and a similar analysis of articular response should be undertaken [27].

The current findings are consistent with studies of treatment response to anti-TNF therapy in adults with RA. It has been reported that males, younger age, lower disease activity measures (i.e. DAS28 and HAQ), concomitant MTX use, no corticosteroid use and fewer previous DMARDs are all associated with good treatment response after at least 6 months of anti-TNF therapy [5–10, 28]. Concomitant MTX use is repeatedly reported to be associated with a good response in adult literature, particularly in studies specifically comparing etanercept and MTX in combination *vs* etanercept alone [7, 8, 29]. This superiority of etanercept and MTX combination has also been noted in the paediatric population (OR 2.1; 95% CI: 1.2, 3.5) [30], particularly in the RF negative JIA population (OR 2.0; P = 0.03) [31]. However, none of the previous studies, nor the current study, have identified concurrent MTX as a predictor of good response in patients treated with etanercept. It is possible that children with JIA may benefit differently to MTX in comparison with RA patients, as has been shown in adults with psoriatic arthritis, where the benefits of concurrent MTX are less clear [32]. Alternatively, a reason for these observed differences may be the vast differences in sample sizes, thus giving varying power to identify factors associated with outcomes. Different outcome measures in RA and JIA may be another reason MTX is not seen as a predictor in paediatric research. However, the benefit in adults with RA have been seen across the spectrum of measures (EULAR response, remission, drug survival), and across the various paediatric studies many outcomes measures have also been investigated. Finally, reasons for the benefit of MTX co-therapy in RA are poorly understood but increasing data may support a role for non-response related to immunogenicity, which may develop differently in JIA (although the data to support this case in adults for etanercept (as opposed to monoclonal antibodies) are less convincing). The Dutch ABC register also identified fewer previous DMARDs to be associated with excellent response in patients treated with etanercept; however, the current study did not have the appropriate data to incorporate this into the model [15].

In various studies investigating efficacy of etanercept according to JIA subtype, systemic JIA has been associated with a poorer response: not achieving ACR Pedi 30 after 1 year (P < 0.01) [16], not achieving ACR Pedi 50 or discontinuing etanercept due to inefficacy by 15 months (P = 0.01) [15], and not achieving ACR Pedi 70 after 6 months (P < 0.001) [14]. Systemic JIA has also been noted to influence the rate of inactive disease: 29.6% in systemic JIA, 54.1% in non-systemic JIA patients [17]. However, in the current study, systemic JIA did not emerge as an independent predictor of response, although there was a weak trend for patients with systemic JIA to have a lower proportion of patients achieving higher ACR Pedi response compared with other subtypes (not statistically significant), but the proportion achieving ACR Pedi 30 responses was similar. While only 73 (15%) patients in this cohort had systemic JIA, this percentage is very similar to the other JIA studies referenced. However, we did not have enough power to conduct a separate analysis in just the systemic JIA cohort. As systemic JIA was not selected in the final model it is possible the study is underpowered to draw any conclusions regarding systemic JIA as a response predictor. The register also did not capture complete details of response among extra-articular manifestations of disease in children with systemic JIA, and therefore, although their joints may have improved to some degree, there may have been other features of disease that did not.

When investigating factors associated with response it is important to differentiate between those that specifically predict response to drug, and those that are prognostic factors for outcome regardless of treatment. Currently, etanercept is the only biologic therapy to be investigated in children with JIA. Therefore, the observed factors of non-response are assumed to be predictors. As more data become available on other biologics (using registers such as the Biologics for Children with Rheumatic Diseases study [33], or ABC [15]), if the same factors predict non-response in these biologics, it may be that they indicate a more resistant disease phenotype rather than anything specific to etanercept. A study has successfully predicted ACR Pedi 70 MTX non-response using multidrug resistance protein 1 [34]. Within the ABC register cohort (n = 153), no association was found between the presence of anti-carbamylated protein antibodies and ACR Pedi 30 response or reaching inactive disease at 15 months after starting anti-TNF treatment [35]. Similar larger studies could be undertaken in children specifically on etanercept to minimize exposure to treatment long-term.

This study was on the largest cohort of JIA patients treated with etanercept in the UK, and it is the second largest study of response in patients with JIA treated with etanercept to be conducted. However, as is the case with most observational studies that obtain data from a real-world clinical setting, there were missing disease activity data at each time point. Multiple imputation was used to account for this. Data on previous DMARDs were not collected and therefore could not be accounted for in the analysis, although a majority of current patients in the UK will proceed directly to a biologic following lack of response to MTX. The duration of follow-up included in this analysis was relatively short (1 year), with respect to disease outcomes. The results cannot be used to comment on longer term sustainability of response. An analysis to investigate longer-term (i.e. 5 years) remission statistics of children, including into adulthood, would be important to inform patients, families and their physicians on longer-term outcomes with therapy.

### Conclusion

In conclusion, among this real-world cohort of children with severe JIA, a significant proportion of children achieved an excellent ACR Pedi and MDA response score within 1 year of starting etanercept, although few clinical factors could predict this outcome. The finding of a greater response in younger children and those with a history of CAU warrants further investigation and may relate to differences in disease phenotype, drug pharmacokinetics or adherence. Despite these positive outcomes, only one child in the study stopped etanercept therapy within 1 year due to remission.

## Supplementary Material

Supplementary Data
