# Supplementary material for: Factors associated with improvement in disease activity following initiation of etanercept in children and young people with Juvenile Idiopathic Arthritis: results from the British Society for Paediatric and Adolescent Rheumatology Etanercept Cohort Study
Source: Rheumatology (Oxford). 2015 Dec 30;55(5):840–7. doi: 10.1093/rheumatology/kev434 (PMC4830911; doi:10.1093/rheumatology/kev434)
Supplement: Supplementary Data [file supp_55_5_840__index.html]

Factors associated with improvement in disease activity following initiation of etanercept in children and young people with Juvenile Idiopathic Arthritis: results from the British Society for Paediatric and Adolescent Rheumatology Etanercept Cohort Study — Factors associated with improvement in disease activity following initiation of etanercept in children and young people with Juvenile Idiopathic Arthritis: results from the British Society for Paediatric and Adolescent Rheumatology Etanercept Cohort Study — Supplementary Data 

# Factors associated with improvement in disease activity following initiation of etanercept in children and young people with Juvenile Idiopathic Arthritis: results from the British Society for Paediatric and Adolescent Rheumatology Etanercept Cohort Study

## Supplementary Data

files

- Supplementary Data - docx file
