# Supplementary material for: Factors associated with improvement in disease activity following initiation of etanercept in children and young people with Juvenile Idiopathic Arthritis: results from the British Society for Paediatric and Adolescent Rheumatology Etanercept Cohort Study
Source: Rheumatology (Oxford). 2015 Dec 30;55(5):840–7. doi: 10.1093/rheumatology/kev434 (PMC4830911; doi:10.1093/rheumatology/kev434)
Supplement: Supplementary Data [file supp_kev434_rhe-15-0542-File002.docx]

**Supplementary Table S1: Four observational studies which looked in-depth at factors associated with response in children with Juvenile Idiopathic Arthritis treated with etanercept.**

| Register / Country | Year | N | Primary Outcome | Time Frame | Analysis Model | Reference |
| --- | --- | --- | --- | --- | --- | --- |
| BiKER, Germany | 2014 | 863 | Achieve ACR Pedi 70 | 6 months | Multivariable logistic regression model:  CHAQ-DI (OR = 0.70 [0.56, 0.88]; p=0.002)  ESR (OR = 1.02 [1.01, 1.03]; p<0.001)  Concomitant Steroids (OR = 0.68 [0.49, 0.94]; p=0.018)  Systemic arthritis (OR = 0.28 [0.16, 0.52]; p<0.001)  Age (OR = 0.94 [0.91, 0.98]; p=0.002)  Area under ROC curve of the model 0.646 | Geikowski T, et al. (2014) [1] |
| Italy | 2013 | 168 | Achieve clinically inactive disease | Median follow-up per patient 2.2 years | Cox proportional hazards regression:  Age at disease onset < 3.6 years (HR = 1.61 [1.04, 2.49]; p=0.03)  Absence of wrist involvement (HR = 2.19 [1.38, 3.48]; p=0.0006) | Solari N, et al. (2013) [2] |
| ABC, Netherlands | 2011 | 262 | Failure to achieve ACR Pedi 50 | 15 months | Multivariable logistic regression:  Females (OR = 2.16 [1.12, 4.18]; p=0.02)  Systemic arthritis (OR 2.92 [1.26, 6.80]; p=0.01)  ANA positive (OR 1.29 [0.66, 2.52]; p=0.47)  Age (OR = 1.08 [0.99, 1.16]; p=0.07)  Disease duration (OR 0.95 [0.87, 1.05]; p=0.31)  Number of DMARDs (OR = 1.21 [0.83, 1.76]; p=0.33)  VAS disease activity by physician (OR 0.95 [0.83, 1.09]; p=0.53)  CHAQ (OR 1.47 [0.98, 2.20]; p=0.07)  ESR (OR 0.99 [0.98, 1.00]; p=0.21) | Otten MH, et al. (2011) [3] |
|  |  |  | Achieve inactive disease | 15 months | Females (OR=0.85 [0.45, 1.59]; p=0.61)  Systemic arthritis (OR 0.49 [0.20, 1.18]; p=0.11)  ANA positive (OR 0.73 [0.37, 1.46]; p=0.38)  Age (OR = 0.92 [0.84, 0.99]; p=0.03)  Disease duration (OR 1.05 [0.96, 1.15]; p=0.26)  Number of DMARDs (OR = 0.64 [0.43, 0.95]; p=0.03)  VAS disease activity by physician (OR 0.89 [0.77, 1.02]; p=0.10)  CHAQ (OR 0.49 [0.33, 0.74]; p=0.001)  ESR (OR 1.03 [0.57, 1.85]; p=0.92) |  |
| France | 2003 | 61 | Failure to achieve ACR Pedi 30 | 12 months | Logistic regression adjusted for age, disease duration, 6 core set for JIA:  Systemic arthritis vs. oligoarticular (p=0.0002)  Systemic arthritis vs. polyarticular (p=0.0031)  Polyarticular vs. oligoarticular (p=0.54) | Quartier P, et al. (2003) [4] |

OR: odds ratio; HR: hazard regression; CHAQ: childhood health assessment questionnaire; VAS: visual analog score

**References**

1 Geikowski T, Becker I, Horneff G, on behalf of the German BRCSG. Predictors of response to etanercept in polyarticular-course juvenile idiopathic arthritis. Rheumatology 2014.

2 Solari N, Palmisani E, Consolaro A, et al. Factors associated with achievement of inactive disease in children with juvenile idiopathic arthritis treated with etanercept. The Journal of rheumatology 2013;40(2):192-200.

3 Otten MH, Prince FH, Armbrust W, et al. Factors associated with treatment response to etanercept in juvenile idiopathic arthritis. Jama 2011;306(21):2340-7.

4 Quartier P, Taupin P, Bourdeaut F, et al. Efficacy of etanercept for the treatment of juvenile idiopathic arthritis according to the onset type. Arthritis and rheumatism 2003;48(4):1093-101.
